# Experimental Determination of the Relationship Between the Resistance Micro-Drilling Characteristic and the Density of Spruce Wood at Different Moisture Contents

**DOI:** 10.3390/ma19143140

**Published:** 2026-07-22

**Authors:** Věra Heřmánková, Ondřej Anton, Kristýna Hrabová, Petr Cikrle, Dalibor Kocáb

**Affiliations:** Faculty of Civil Engineering, Brno University of Technology, Veveří 331/95, 602 00 Brno, Czech Republic; vera.hermankova@vut.cz (V.H.); ondrej.anton@vut.cz (O.A.); petr.cikrle@vut.cz (P.C.); dalibor.kocab@vut.cz (D.K.)

**Keywords:** wood density, wood compressive strength, timber structure diagnostics, resistance drilling, non-destructive methods

## Abstract

This study explores the potential of non-destructive methods for diagnosing timber structures, with a primary focus on maximising the capabilities of the resistance-drilling technique. Laboratory tests were performed on spruce wood specimens, the most commonly used construction timber in Central Europe, prepared across a wide range of moisture contents (0–53%) to assess the influence of moisture on resistance-drilling characteristics. The resistance micro-drilling (RM) characteristic was found to be independent of moisture content (coefficient of determination close to zero), confirming that resistance drilling provides stable results under varying in situ moisture conditions. In contrast, wood density and the RM characteristic were strongly correlated, with coefficients of determination of R^2^ = 0.87 for moisture contents between 0% and 30%, and R^2^ = 0.90 for the 8–18% moisture range typical of timber in service. Based on these relationships, two linear conversion equations were developed (ρ = 1.685·RM + 183.85 and ρ = 1.982·RM + 135.15, respectively), enabling estimation of spruce wood density directly from RM values. Compressive strength parallel and perpendicular to grain decreased with increasing moisture content up to the fibre saturation point, beyond which the reduction in strength plateaued.

## 1. Introduction

Wood is among the longest-used construction materials and forms one of the fundamental components of the world’s architectural heritage. At the same time, it represents a highly promising, renewable, and environmentally sustainable material suitable for modern construction applications [1]. Its use in new buildings as well as interventions in existing timber structures therefore requires a detailed understanding of its material characteristics and structural behaviour. This, however, is relatively complex due to the nature of wood as a biodegradable, hygroscopic, and strongly anisotropic material, and also due to the lack of clear methodological guidance in the currently valid standards and regulations [2].

The diagnostics of timber structures is a relatively young discipline whose theoretical and instrumental foundations stem from methods originally developed for assessing standing trees. Many of the instruments and procedures used today were initially created specifically for evaluating the quality of living wood. For a long time, research focused primarily on historic structures and on wood species used in the past, especially within the context of heritage conservation. In contemporary engineering practice, however, the need for detailed assessment of modern structures—predominantly constructed from spruce wood—is steadily increasing. For this reason, current research is largely oriented toward the physical and mechanical behaviour of Norway spruce [3].

For the diagnostics of timber structures, it is essential to determine two fundamental parameters required for structural assessment:

- The presence and extent of biotic degradation (wood-destroying insects, decay fungi, microscopic moulds);
- The current physical and mechanical properties of the material.

Both of these factors are closely linked to the moisture condition of the wood.

Wood, as a porous and hygroscopic natural material, accumulates water in both the cell walls and the macro-void structure. The quantity of water is expressed as moisture content (the ratio of water mass to dry mass). This value strongly depends on temperature and relative humidity. Within the hygroscopic range (0–97/98% relative humidity), sorption occurs primarily into the cell walls. At extremely high relative humidity (>98%), capillary condensation inside the cell cavities begins to dominate. A key material parameter is the fibre saturation point (FSP), above which further increases in moisture no longer significantly affect the physical properties of wood [4].

The FSP value is species-specific: approximately 22–24% for oak and ash, 26–28% for pine and larch, and typically 30–34% for fir and spruce [5]. Variations in moisture below the fibre saturation point have a major impact on the physical, mechanical, and rheological behaviour of wood, including shrinkage, swelling, strength, stiffness, and modulus of elasticity [4,5,6].

Moisture therefore significantly affects all key material properties. Elevated water content additionally creates conditions favourable for biological degradation of wood. Correct identification of the moisture state is thus a fundamental prerequisite for the design, execution, and long-term maintenance of timber structures. This corresponds with the increasing importance of long-term in situ moisture monitoring of structural elements [7].

Biotic degradation can occur only when the moisture content of the wood is sufficiently high. Standard EN 335 [8] classifies wood into five use classes according to its moisture condition and exposure. In simplified terms, at moisture below 10%, biological attack is not expected; between 10 and 20%, insect attack may occur; above 20%, the development of decay fungi and mould becomes increasingly likely.

Fungal degradation leads to reduced density, changes in physical and mechanical parameters, and decreased stiffness and strength, and is typically spatially localised. In contrast, insect-related degradation is generally more diffuse, characterised by an extensive tunnel system and greater spatial variability of damage [9]. Numerous experimental studies have confirmed a strong correlation between density loss and the mechanical properties of insect-damaged wood, which supports the use of residual density measurements as an indicator of the remaining strength of structural elements. Nevertheless, further development and standardisation of diagnostic methods suitable for practical application is still necessary [9,10].

Both qualitative and quantitative assessment of insect-related degradation, on the surface as well as within the depth of timber members, are essential for determining the effective cross-section and therefore the safety of the entire structure [11].

Although the relationship between resistance-drilling characteristics and wood density has been investigated for several species, a systematic, quantitative evaluation of this relationship for spruce wood—the dominant construction timber in Central Europe—across the full range of moisture contents relevant to existing structures, including above the fibre saturation point, is still lacking. The novelty of this study lies in the derivation of moisture range-specific conversion equations between the RM resistance-drilling characteristic and wood density, together with an explicit verification that this relationship remains stable irrespective of moisture content. The aim of this study is therefore to experimentally determine the relationship between the RM characteristic, density and compressive/bending strength of spruce wood under varying moisture conditions, and to propose practical equations for converting RM values into estimates of density for the diagnostics of existing timber structures. To achieve this aim, the following tasks were addressed: (1) determination of density, RM characteristic and compressive strength (parallel and perpendicular to grain) for spruce specimens conditioned across a wide moisture range (Experiment A); (2) verification of the RM–density relationship at a bending-relevant moisture range and specimen geometry (Experiment M); and (3) derivation and validation of linear conversion equations between RM and density for the 0–30% and 8–18% moisture ranges.

## 2. Diagnostics of Timber Structures

### 2.1. Visual Assessment

Visual assessment represents the primary diagnostic procedure that enables rapid identification of macroscopic defects and structural anomalies. This method is essential for initiating a detailed inspection; however, its diagnostic reliability is limited by operator subjectivity and the absence of unified standards. Visually detectable indicators of biotic degradation include the presence of exit holes, frass deposits near the damaged area, discoloration of the wood, and possible fruiting bodies of decay fungi.

Tracing signs of water ingress makes it possible to approximate the moisture history of the element, which is crucial for predicting biotic processes. In areas with restricted accessibility, endoscopic optics are used, significantly increasing the information capacity of visual diagnostics [12].

### 2.2. Moisture

The moisture condition is a dominant factor influencing the physical–mechanical and biological parameters of wood. Its accurate determination is therefore an essential prerequisite for any material analysis. In situ, resistance-type moisture meters with driven electrodes are most commonly used; their measurements are particularly reliable in the range of approximately 5–25%. Proper interpretation requires consideration of the wood species, temperature, and local heterogeneity.

The methodological framework for this procedure is defined in standard EN 13183-2 [13]. The moisture distribution within a structural element is generally spatially variable, and therefore multiple measurements must be taken to obtain a representative characterisation of the material.

### 2.3. Non-Destructive Methods for Assessing In-Service Timber

Non-destructive testing (NDT) methods constitute a key group of diagnostic procedures that allow the assessment of mechanical properties, density, and the extent of biotic degradation without damaging the element. Technical Committee of RILEM, 215-AST formalises methodological recommendations for selected non-destructive and semi-destructive techniques, including visual inspection, moisture measurement, species identification, digital radioscopy, and ground-penetrating radar surveys [14,15]. A comprehensive overview of NDT techniques is also provided in [16].

Research focused on historic structures—particularly chestnut timber [17,18,19,20,21,22,23]—has demonstrated the high potential of NDT methods for estimating mechanical parameters through their correlation with density. Study [20] shows that once density has been determined, compressive and bending strength characteristics can be reliably approximated. Additional applications of NDT in historically exposed structures are presented, for example, in [24].

#### 2.3.1. Resistance Pin Penetration

One commonly used method is resistance pin penetration. The Pilodyn 6J instrument is most frequently used; it is a mechanical device that measures the penetration depth of a steel pin under a constant impact energy. Due to its limited penetration depth (max. 40 mm), this method provides only near-surface information on wood density and structure. Measurements are most reliable in the radial direction, where the alternating earlywood and latewood layers result in a more stable mechanical response. The values obtained can be used to estimate wood density at 12% moisture [12,25]. The correlation between penetration depth and mass loss—particularly in biologically degraded samples—has been experimentally confirmed in [24]. However, the results are sensitive to local defects and to the depth limitations of the method.

#### 2.3.2. Resistance Micro-Drilling

Resistance micro-drilling (or only resistance drilling) is one of the most important in situ methods for assessing internal wood structure. Measuring mechanical resistance at a constant drilling speed provides a detailed density profile and enables the detection of wood degradation (decay, insect damage) as well as natural wood features (cracks, knots, and annual-ring transitions) [12,26,27,28]. Recent applications of the method to historic timber structures include the estimation of wood density in dismantled roof members of a late-19th-century building, combined with stress-wave velocity to derive the dynamic modulus of elasticity for in situ assessment [29].

**Figure 1:**
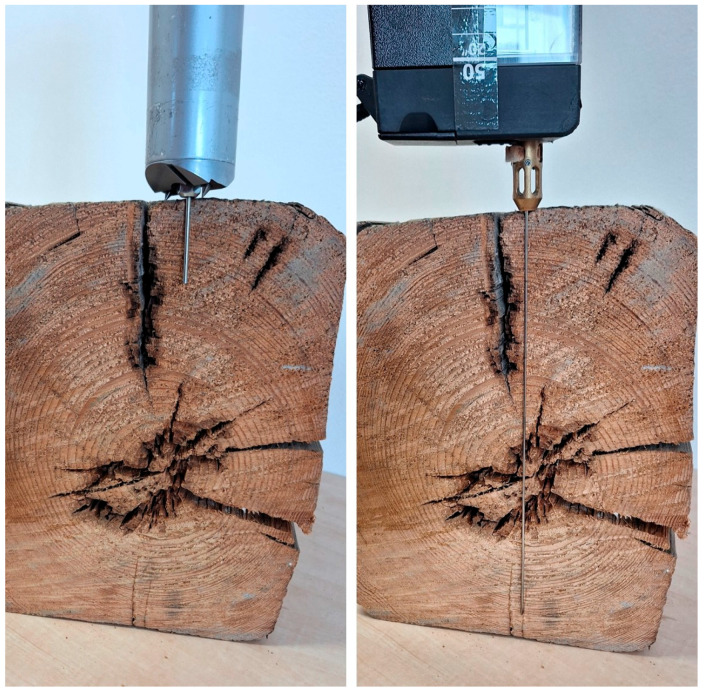
Difference in penetration depth of measurement methods.

The instrument commonly used for resistance drilling is the Resistograph, which enables drilling to depths up to 500 mm, significantly exceeding the penetration depth achievable with the Pilodyn 6J (see Figure 1).

**Figure 2:**
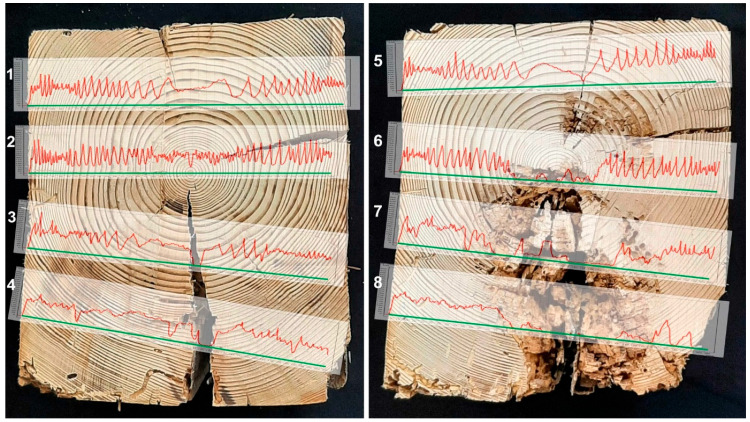
Differences in drilling-resistance records in fir wood—the green line represents the drilling path, the red curve shows the measured resistance; 1—off-centre drilling; 2—radial drilling; 3—off-centre drilling, reduced resistance due to a crack; 4—off-centre drilling, reduced resistance due to a crack and insect exit holes; 5—off-centre drilling, reduced resistance due to a small crack; 6, 7 and 8—reduced resistance caused by decay-related wood degradation.

The method provides an immediate graphical output as well as a digital record that can be subsequently analysed. The horizontal axis represents the drilling depth, and the vertical axis shows the energy required to maintain a constant drilling speed. Higher drilling resistance corresponds to higher wood density. The alternating higher and lower resistance values are caused by the natural alternation of latewood and earlywood. Figure 2 shows examples of drilling-resistance traces, representing the most common types of recorded profiles.

The Resistograph device was originally developed for studying the anatomy of various wood species, for evaluating wood quality, assessing tree-growth rates, and ensuring the operational safety of standing trees [27,30]. It is also used, for example, to determine the density of eucalyptus wood [31].

Feio investigated the correlation between mechanical properties and NDT methods [21,22,32] and in [33] defined the RM drilling-resistance characteristic as

(1) $$RM=\frac{S}{h},$$

where *RM* is the resistance characteristic (its unit depends on the type of Resistograph used), *S* is the area under the curve (its unit likewise depends on the device type), and *h* is the length of the measured segment. All calculations and statistical analyses were performed in Python 3.11 (Python Software Foundation).

This characteristic makes it possible to quantify the material’s response and compare it with density or other physical–mechanical parameters of wood.

Several studies [33,34,35,36,37] confirm a strong correlation between resistance values and wood density, while the influence of moisture appears marginal in some cases [37]. Conversely, studies [38,39,40] report that the correlation can be variable and inconclusive, highlighting species specificity and the importance of careful calibration. Recent studies using optimised drilling parameters report even stronger RM–density correlations, with coefficients of determination of up to 0.97–0.99 [41,42], and the drilling-resistance signal has likewise been linked to wood micro-density [43]. By contrast, the relationship between drilling resistance and mechanical strength is consistently reported as weaker and considerably more variable: correlations have been described as only “acceptable” for chestnut wood [44], no statistically significant correlation was found once natural defects were present in real structural elements [45], and the relationship is reported to be strongly conditioned by confounding factors such as wood species, drilling direction and moisture content [46].

#### 2.3.3. Comparison of Pilodyn and Resistograph Test Results

Both methods use the resistance principle; however, their information capacity differs fundamentally. The Pilodyn provides a surface-level, quick and simple density estimate, whereas the Resistograph offers a detailed depth profile, including detection of internal degradation that the Pilodyn cannot capture.

In diagnostic practice, the two methods can be combined: the Resistograph is used for in-depth structural analysis, while the Pilodyn may serve as a rapid preliminary density calibration tool, as suggested in [25]. Nevertheless, this combination is only temporary, as the Pilodyn is no longer manufactured. Moreover, it has been shown that the Resistograph provides a more reliable and less variable density estimate than the Pilodyn [47].

## 3. Materials and Methods

The aim of the experiment was to analyse the relationships between the RM resistance-drilling characteristic, density, and the mechanical properties of spruce wood across a wide range of moisture contents. The experiment was divided into two parts:

- Experiment A: compressive strength perpendicular/parallel to grain, density, and RM characteristics;
- Experiment M: compressive strength and bending strength, density, and RM characteristics.

### 3.1. Experiment A

In Experiment A, the following wood properties were determined:

- Absolute moisture content;
- Density;
- Resistance drilling measurement—RM characteristics;
- Compressive strength parallel to grain $f_{c,\|,u}$;
- Compressive strength perpendicular to grain $f_{c,⊥,u}$

The spruce (*Picea abies*) timber used for the experiment was a single batch of six beams with a nominal cross-section of 120 mm × 160 mm and a length of 2000 mm. Test specimens were subsequently cut from these beams using a table circular saw. Six sets of spruce wood specimens (A-A to A-F), each containing 20 pieces without macroscopic defects (knots, resin pockets, decay, or signs of biotic degradation), were selected for the experiment. Within each set, all specimens were taken from a single piece of timber to minimise spatial variability.

Each set contained:

- Ten specimens of type ⟂ (nominal dimensions 55 mm × 90 mm × 110 mm), intended for measuring compressive strength perpendicular to grain $f_{c,⊥,u}$ and RM characteristics;
- Ten specimens of type ∥ (nominal dimensions 55 mm × 55 mm × 340 mm), intended for measuring compressive strength parallel to grain $f_{c,\|,u}$.

The specimens were first oven-dried at (103 ± 2) °C until the difference between two consecutive mass measurements was less than 0.1%. Immediately after removal from the oven, their masses (accuracy 0.01 g) and dimensions (accuracy 0.01 mm) were recorded.

For set A-A, the planned tests were conducted immediately, i.e., on specimens with 0% moisture content. The remaining sets (A-B to A-F) were conditioned to different moisture contents either in a climate chamber (A-B to A-D) or in a water bath at (20 ± 2) °C (A-E and A-F).

For the conditioned sets A-B to A-F, moisture content after testing was determined according to standard EN 13183-1 [48]. The results are presented in Table 1. The density of all specimens was calculated from their measured masses and dimensions according to standard EN 384+A2 [49]. The results are presented in Table 2.

**Figure 3:**
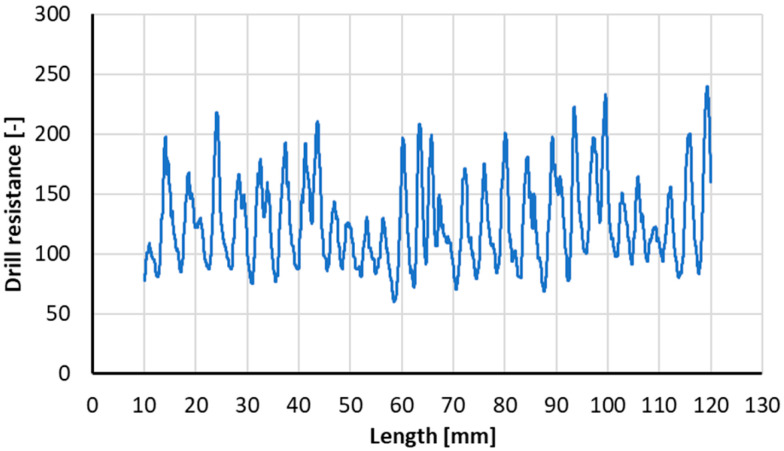
Example of a drilling record prepared for the calculation of the area under the curve.

For resistance micro-drilling, a Rinntech Resistograph R650-EA device (RINNTECH GmbH, Heidelberg, Germany) was used. The RM characteristic was determined on specimens from sets A-A-⟂ to A-F-⟂. The specimens were tested in the radial direction, and each specimen was drilled three times. All drilling curves used for calculating the area under the curve were trimmed to the interval 10–120 mm to eliminate the influence of edge effects. Figure 3 shows an example of a typical drilling record. The determined RM resistance-drilling characteristics are presented in Table 3.

**Figure 4:**
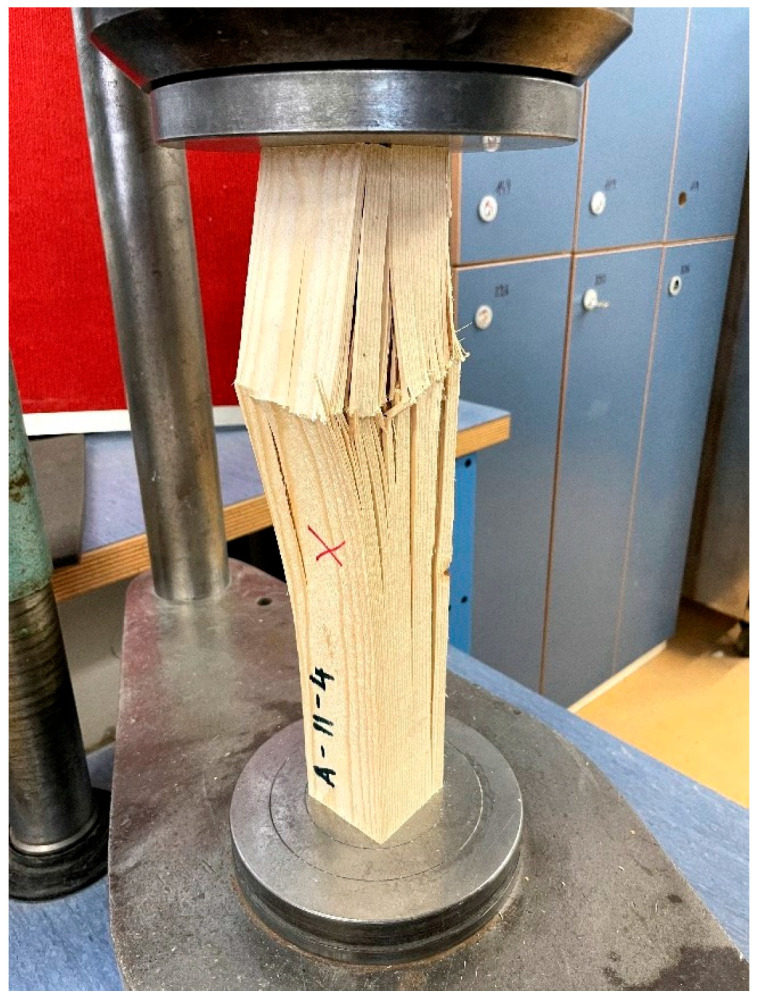
Determination of compressive strength parallel to grain.

The compressive strength perpendicular to grain $f_{c,⊥,u}$ was determined on specimens A-A-⊥ to A-F-⊥ in accordance with standard EN 408+A1 [50]. The force $F_{c,⊥,max}$ was obtained iteratively from the load–displacement diagram. The results are presented in Table 4. The compressive strength parallel to grain $f_{c,\|,u}$ was determined on specimens A-A-∥ to A-F-∥, also in accordance with standard [50]. The test setup is documented in Figure 4, and the results are shown in Table 5.

### 3.2. Experiment M

In the second partial experiment, two sets of test specimens (M-A and M-B) were conditioned to reach moisture contents close to the reference value (~12%). The following tests were carried out on the spruce wood:

- M-A: bending strength parallel to grain $f_{m,u}$;
- M-B: compressive strength parallel to grain $f_{c,\|,u}$;
- Both sets: absolute moisture content, density, resistance drilling measurement—RM characteristic.

Both sets consisted of eight test specimens that showed no visible signs of damage. The specimens of set M-A had nominal dimensions of 25 mm × 45 mm × 850 mm, and those of set M-B had nominal dimensions of 25 mm × 45 mm × 150 mm. The specimens were prepared from beams with a length of 2000 mm.

For all specimens, moisture content was determined according to [48] and density according to [49], in the same way as in Experiment A. The results are presented in Table 6.

**Figure 5:**
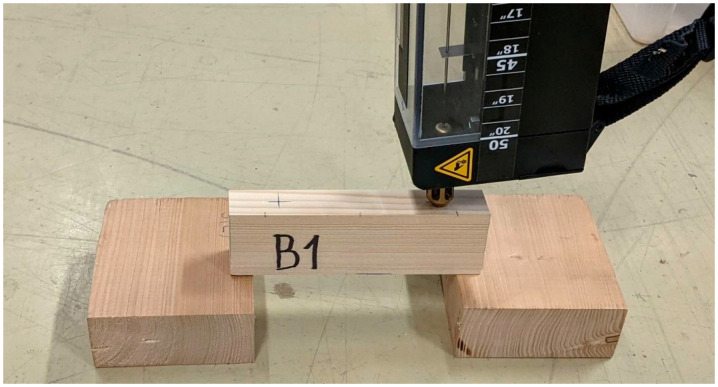
Resistance micro-drilling on a specimen of set M-B.

**Figure 6:**
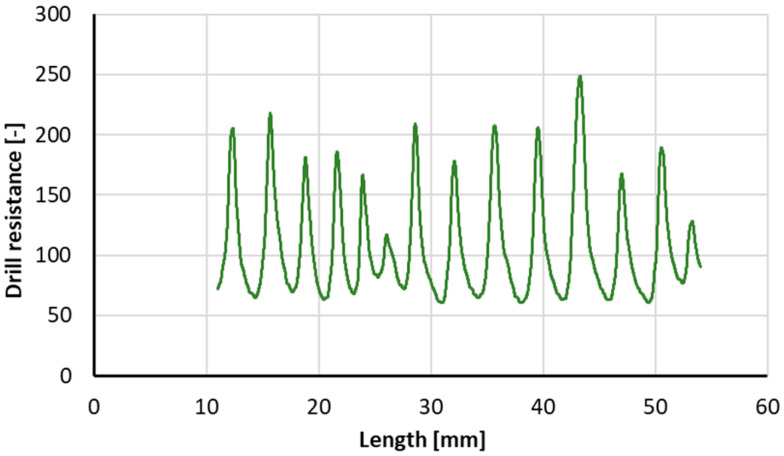
Example of a drilling record prepared for the evaluation of the area under the curve.

Resistance micro-drilling using the Rinntech Resistograph R650-EA was performed six times on all specimens of set M-A and twice on all specimens of set M-B. All drilling graphs used for calculating the area under the curve were limited to the interval 11–54 mm to ensure that the results were not affected by edge effects. Figure 5 documents the measurement performed with the Resistograph, and Figure 6 shows an example of a typical drilling record. The determined RM drilling-resistance characteristics are presented in Table 7.

**Figure 7:**
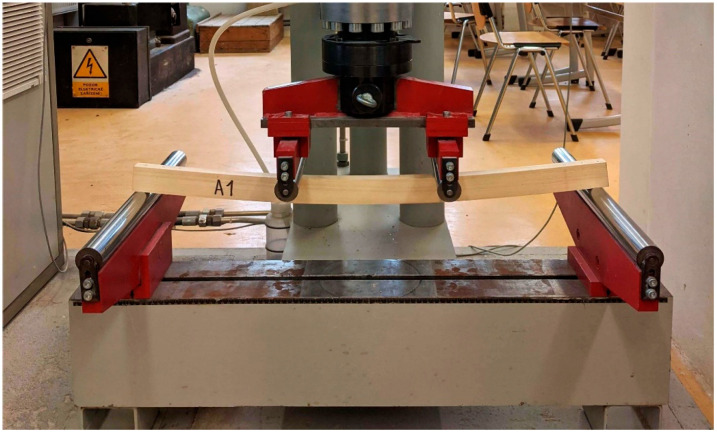
Determination of bending strength parallel to grain.

For all specimens of set M-A, the bending strength parallel to grain $f_{m,u}$, was determined (see Figure 7). For the specimens of set M-B, the compressive strength parallel to grain $f_{c,\|,u}$ was determined. Both tests were carried out in accordance with [50], and the results are presented in Table 8. The obtained values correspond to the typical characteristics of spruce wood under conditions close to 12% moisture content.

## 4. Results and Discussion

The measured data confirmed the moisture-dependent changes in wood properties described in publications [4,5,6,7]. It was also verified that the properties of wood differ depending on whether the fibre saturation point (FSP) has been reached.

**Figure 8:**
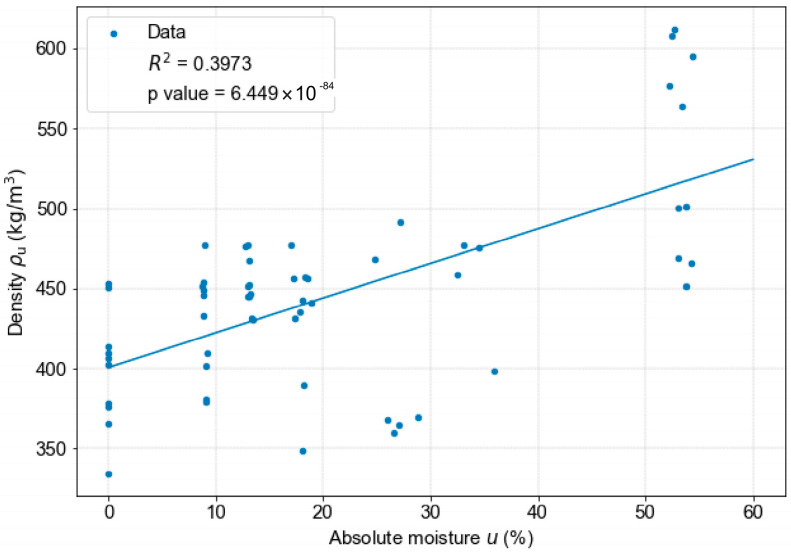
Relationship between wood density and moisture content (sets A-A-⊥ to A-F-⊥).

**Figure 9:**
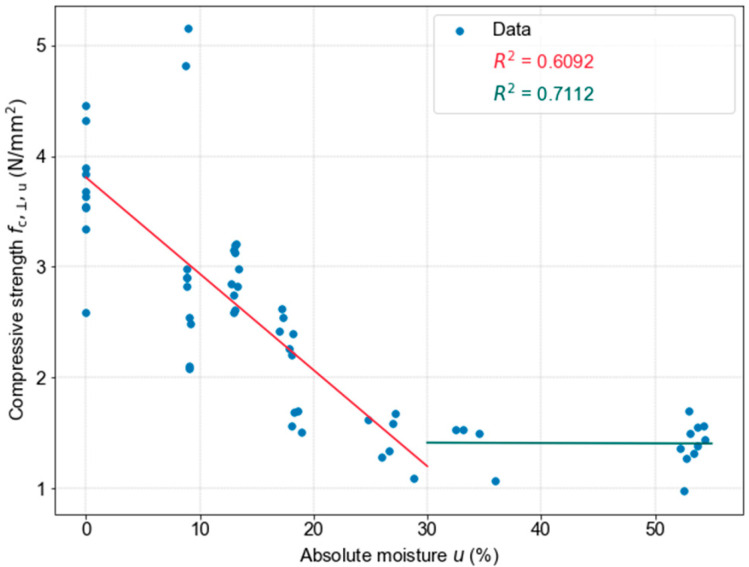
Relationship between compressive strength perpendicular to grain and moisture content (sets A-A-⊥ to A-F-⊥).

**Figure 10:**
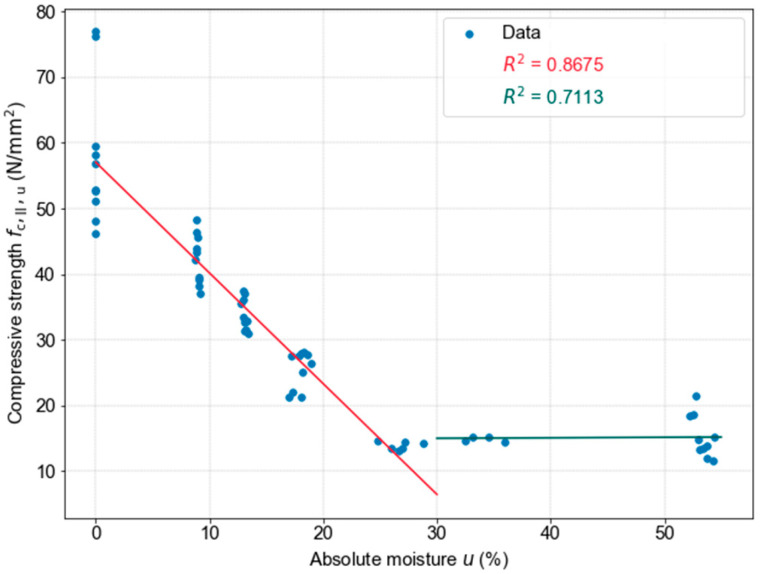
Relationship between compressive strength parallel to grain and moisture content (sets A-A-∥ to A-F-∥).

Figure 8 shows the relationship between wood density and its moisture content. In this case, reaching the FSP did not produce any pronounced change in the data trend. Figure 9 presents the relationship between compressive strength perpendicular to grain and moisture content, and Figure 10 presents the relationship between compressive strength parallel to grain and moisture content. In both graphs (Figure 9 and Figure 10), trendlines were added to the experimental data—one for moisture content up to approximately 30% and one for moisture content above 30%. In both cases, once the FSP is reached, the reduction in strength plateaus and the values remain at comparable levels.

**Figure 11:**
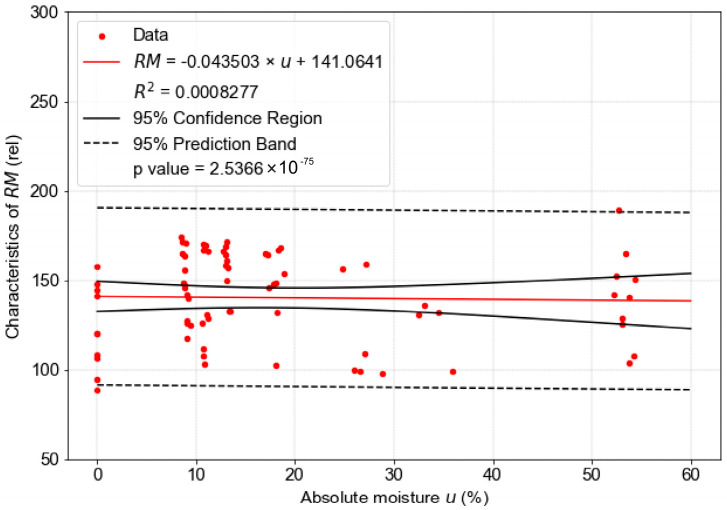
Relationship between RM characteristic and moisture content (sets A-A-⊥ to A-F-⊥, M-A, M-B).

Figure 11 shows the relationship between the RM characteristic and wood moisture content. The graph includes all RM measurements from both partial experiments A and M. The analysis shows that the RM characteristic is independent of moisture content (the coefficient of determination R^2^ is close to zero). Considering that resistance drilling is used as an in situ diagnostic method, this is a favourable finding.

All RM values used in the regression models were obtained from radial drilling measurements.

**Figure 12:**
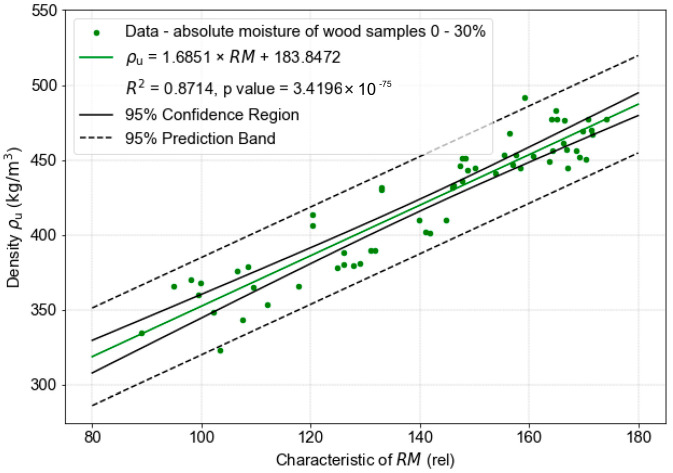
Relationship between density and RM characteristic (sets A-A-⊥ to A-F-⊥, M-A, M-B with moisture 0–30%).

Next, the relationship between wood density and the RM characteristic was determined. This relationship was evaluated only for moisture content between 0% and 30%. The reason is twofold: strength values do not change once the FSP is reached (see Figure 9 and Figure 10), and such high moisture content occurs in structures only in exceptional circumstances. The resulting graph (Figure 12) shows the dependence of density on the RM characteristic for moisture content below the fibre saturation point. A strong correlation can be observed, with a coefficient of determination R^2^ = 0.87, indicating that the linear model

(2) $$\rho_{u;0-30\%}=1.685\cdot RM+183.85,$$

provides a good fit to the experimental data.

**Figure 13:**
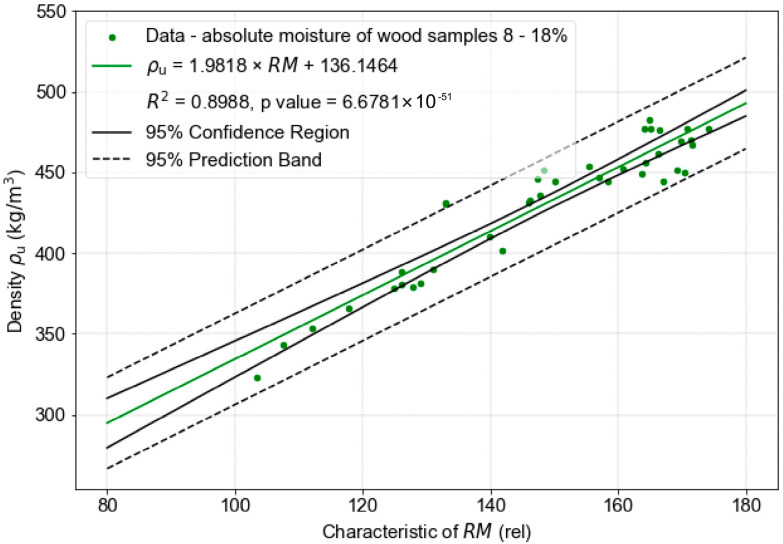
Relationship between density and RM characteristic (sets A-A-⊥ to A-F-⊥, M-A, M-B with moisture 8–18%).

Standard [49] allows the conversion of density measured at moisture content within the interval 8% ≤ u ≤ 18% to the reference density at 12% moisture. Therefore, a second graph was constructed (Figure 13), showing the dependence of density on the RM characteristic only for specimens with moisture content in the 8–18% range. Again, a strong correlation is evident, with a coefficient of determination R^2^ = 0.90. This indicates that the linear model:

(3) $$\rho_{u;8-18\%}=1.982\cdot RM+135.15,$$

also explains the experimental data well.

Thus, RM characteristic values can be converted into wood density, and subsequently, according to the moisture-conversion rules given in EN 384+A2 [49], into the reference density at 12% moisture.

The RM–density correlations obtained in this study (R^2^ = 0.87 for the 0–30% moisture range and R^2^ = 0.90 for the 8–18% range) are consistent with, though somewhat lower than, the near-perfect correlations reported by Gendvilas et al. (2024a, 2024b) [41,42] for softwood species under optimised drilling parameters (R^2^ = 0.97–0.99), and are comparable to values reported elsewhere in the literature for spruce and other softwoods. They are, however, markedly stronger than the RM–density correlations reported for structurally compromised or defect-containing timber, such as those found by Piazza and Riggio (2008) [45] and Feio, Lourenço and Machado (2005) [44] (R^2^ ≈ 0.5 or lower), which the authors attribute to the use of clear, defect-free specimens under controlled laboratory conditions in the present study. The finding that the RM characteristic is independent of moisture content (R^2^ close to zero, Figure 11) corroborates similar observations elsewhere in the resistance-drilling literature, and supports the conclusion of Íñiguez-González et al. (2015) [46] that drill resistance is comparatively robust to moisture variation relative to other non-destructive parameters.

## 5. Conclusions

This study confirmed a strong correlation between the RM resistance-drilling characteristic and the density of spruce wood. For moisture contents up to 30%, density can be reliably estimated from RM values using linear models, with the highest accuracy in the service-moisture range of 8–18%. The RM characteristic proved to be independent of moisture content, making resistance drilling a stable and robust tool for in situ assessment. In contrast, compressive strengths parallel and perpendicular to grain decreased with rising moisture until reaching the fibre saturation point, beyond which the reduction in strength plateaued. Overall, resistance drilling provides a practical and moisture-insensitive method for estimating density and supporting structural evaluation of existing timber elements.

## Figures and Tables

**Table 1:** The absolute moisture content of the individual test sets in %.

Set	Average Value	Median	Standard Deviation SD	Coefficient of Variation CoV (%)
A-A-ꓕ	0.0	0.0	0.0	0.0
A-B-ꓕ	9.0	8.9	0.1	1.6
A-C-ꓕ	13.1	13.1	0.2	1.3
A-D-ꓕ	18.0	18.1	0.6	3.2
A-E-ꓕ	29.6	28.0	3.8	12.8
A-F-ꓕ	53.3	53.3	0.7	1.3
A-A-‖	0.0	0.0	0.0	0.0
A-B-‖	7.0	6.9	0.3	4.9
A-C-‖	10.3	10.4	0.3	2.8
A-D-‖	16.6	16.7	0.5	2.9
A-E-‖	27.4	27.4	2.8	10.3
A-F-‖	49.6	50.8	3.6	7.3

**Table 2:** The timber density of the individual test sets in kg/m^3^.

Set	Average Value	Median	SD	CoV (%)
A-A-ꓕ	399	404	35	9
A-B-ꓕ	428	439	32	7
A-C-ꓕ	452	449	16	3
A-D-ꓕ	434	442	36	8
A-E-ꓕ	423	429	52	12
A-F-ꓕ	534	533	60	11
A-A-‖	401	399	35	9
A-B-‖	427	434	32	7
A-C-‖	442	444	16	4
A-D-‖	438	442	36	8
A-E-‖	420	419	43	10
A-F-‖	522	514	58	11

**Table 3:** The values of RM characteristic (relative)—experiment A.

Set	Average Value	Median	SD	CoV (%)
A-A-ꓕ	123	120	22	18
A-B-ꓕ	147	147	13	9
A-C-ꓕ	156	160	13	8
A-D-ꓕ	150	151	19	13
A-E-ꓕ	122	120	23	19
A-F-ꓕ	141	141	25	17

**Table 4:** Compressive strength perpendicular to grain in N/mm^2^.

Set	Average Value	Median	SD	CoV (%)
A-A-‖	3.68	3.66	0.49	13.4
A-B-‖	3.08	2.86	1.00	32.6
A-C-‖	2.93	2.91	0.23	7.7
A-D-‖	2.09	2.24	0.41	19.5
A-E-‖	1.42	1.51	0.21	14.5
A-F-‖	1.41	1.41	0.19	13.3

**Table 5:** Compressive strength parallel to grain in N/mm^2^.

Set	Average Value	Median	SD	CoV (%)
A-A-ꓕ	57.8	54.8	10.2	17.6
A-B-ꓕ	42.4	42.8	3.6	8.5
A-C-ꓕ	33.9	33.2	2.3	6.8
A-D-ꓕ	25.5	27.0	2.7	10.7
A-E-ꓕ	14.3	14.4	0.7	4.7
A-F-ꓕ	15.3	14.4	3.0	19.9

**Table 6:** The absolute moisture content and density of timber.

Property	Set	Average Value	Median	SD	CoV (%)
Absolute moisture content [%]	M-A	9.5	9.2	0.9	9.8
	M-B	10.9	10.9	0.2	1.9
Density [kg/m^3^]	M-A	438	449	58	13
	M-B	438	445	50	11

**Table 7:** The values of the RM characteristic (relative)—experiment M.

Set	Average Value	Median	SD	CoV (%)
M-A	146	145	30	20
M-B	144	149	25	18

**Table 8:** The timber strength parallel to grain in N/mm^2^.

Property (Set)	Average Value	Median	SD	CoV (%)
$f_{m,u}$ (M-A)	64.3	70.7	22.4	34.9
$f_{c,\|,u}$ (M-B)	35.4	35.7	5.2	14.7

## Data Availability

The original contributions presented in this study are included in the article. Further inquiries can be directed to the corresponding author.
